# Metabolite Profiles Reveal Energy Failure and Impaired Beta-Oxidation in Liver of Mice with Complex III Deficiency Due to a BCS1L Mutation

**DOI:** 10.1371/journal.pone.0041156

**Published:** 2012-07-19

**Authors:** Heike Kotarsky, Matthias Keller, Mina Davoudi, Per Levéen, Riitta Karikoski, David P. Enot, Vineta Fellman

**Affiliations:** 1 Department of Pediatrics, Clinical Sciences, Lund University, Lund, Sweden; 2 Neonatology, Department of Pediatrics, University Hospital, Essen, Germany; 3 Central Pathology Laboratory, Helsinki University Hospital Laboratory Diagnostics, Helsinki, Finland; 4 Children’s Hospital, Helsinki University Hospital and University of Helsinki, Helsinki, Finland; 5 Folkhälsan Research Center, Helsinki, Finland; University Nijmegen Medical Centre, Netherlands

## Abstract

**Background & Aims:**

Liver is a target organ in many mitochondrial disorders, especially if the complex III assembly factor BCS1L is mutated. To reveal disease mechanism due to such mutations, we have produced a transgenic mouse model with c.232A>G mutation in *Bcs1l*, the causative mutation for GRACILE syndrome. The homozygous mice develop mitochondrial hepatopathy with steatosis and fibrosis after weaning. Our aim was to assess cellular mechanisms for disease onset and progression using metabolomics.

**Methods:**

With mass spectrometry we analyzed metabolite patterns in liver samples obtained from homozygotes and littermate controls of three ages. As oxidative stress might be a mechanism for mitochondrial hepatopathy, we also assessed H_2_O_2_ production and expression of antioxidants.

**Results:**

Homozygotes had a similar metabolic profile at 14 days of age as controls, with the exception of slightly decreased AMP. At 24 days, when hepatocytes display first histopathological signs, increases in succinate, fumarate and AMP were found associated with impaired glucose turnover and beta-oxidation. At end stage disease after 30 days, these changes were pronounced with decreased carbohydrates, high levels of acylcarnitines and amino acids, and elevated biogenic amines, especially putrescine. Signs of oxidative stress were present in end-stage disease.

**Conclusions:**

The findings suggest an early Krebs cycle defect with increases of its intermediates, which might play a role in disease onset. During disease progression, carbohydrate and fatty acid metabolism deteriorate leading to a starvation-like condition. The mouse model is valuable for further investigations on mechanisms in mitochondrial hepatopathy and for interventions.

## Introduction

The cytochrome bc1 complex (complex III, CIII) is composed of one mitochondrial and ten nuclear encoded proteins that are assembled with the help of two chaperones, BCS1L and TTC19. The recently identified protein TTC19 aids assembly of early subunits of CIII [1], whereas BCS1L finalizes it by inserting the Rieske iron-sulfur protein (RISP) and QCR10P into the precomplex [2], [3], [4]. CIII deficiencies have been reported originating from mutations in the mitochondrial encoded cytochrome b, the nuclear encoded subunit VII, BCS1L and TTC19 [5], [6]. More than 20 pathogenic human *BCS1L* mutations have been causative for CIII assembly defects that result in a broad spectrum of symptoms ranging from congenital deafness [7], and myopathy [8], to neonatal encephalopathy [1], [9] or liver disorder usually with proximal tubulopathy [10], [11]. The most severe phenotype, the autosomal recessive GRACILE syndrome (MIM 603358) is caused by a missense mutation (c.232A>G) substituting serine for glycine (p.S78G) in the N-terminus of the protein. The syndrome presents with fetal growth restriction, aminoaciduria, cholestasis, iron accumulation in the liver, and lactacidosis, and results in early death within days or weeks [11], [12].

To elucidate functions of BCS1L in general, and the pathophysiologic mechanisms in GRACILE syndrome in particular, we introduced the *Bcs1l* c.232A>G mutation into mice [13]. The main findings in the homozygous (*Bcs1l* ^G/G^) mice mimick the human disorder and consist of growth failure from the fourth week of live, progressive liver disorder with glycogen depletion and microvesicular steatosis, proximal tubulopathy, and death before 6 weeks [13]. In mitochondria of all tissues, decreased BCS1L protein amount is associated with a deficient assembly of RISP into CIII, resulting in a decreased CIII activity. As in GRACILE patients, liver is the main affected organ and deteriorates quickly in *Bcs1l* ^G/G^ mice.

The pathophysiologic mechanisms underlying the different BCS1L disorders are unclear. However, in neonatal diseases hepatopathy is a common finding. In this study, our aim was to use the transgenic *Bcs1l* ^G/G^ mice to assess changes in liver function at disease onset as well as at the end stage. Targeted metabolite profiles in homogenized liver tissues from the animals was performed in order to get a comprehensive picture of the effect of the homozygous *Bcs1l* c.232A>G mutation on liver metabolism. Compromised respiratory chain function may impair beta-oxidation resulting in fatty acid accumulation and steatosis [14] that further hamper the redox state of the respiratory chain thereby causing oxidative stress [15]. We hypothesized that oxidative stress might contribute to the rapid deterioration in the liver of *Bcs1l* ^G/G^ mice and therefore assessed proposed [7], [16], [17] effects of reactive oxygen species (ROS) as well as antioxidant capacity of liver tissue.

## Results

### Effect of Genotype and Age on Metabolite Profiles

**Figure 1:**
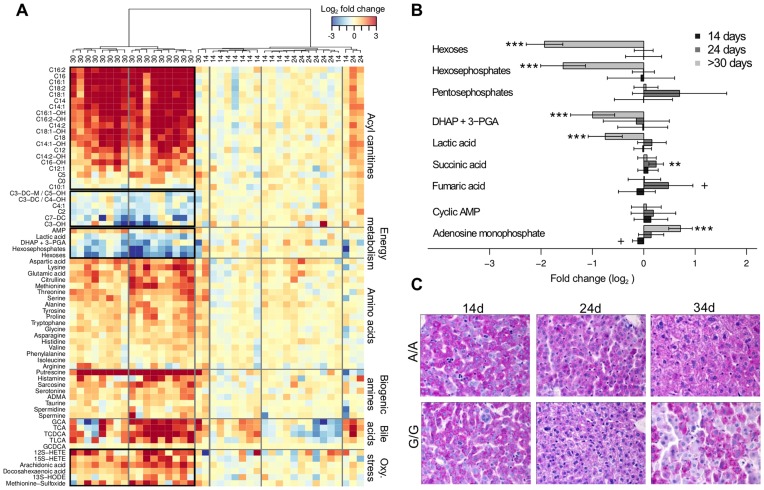
Carbohydrate metabolism. (A) Heatmap depicting the log basis 2 fold changes in metabolite (rows) concentration determined in liver tissues between matched pairs (columns) of homozygous (G/G, *Bcs1l* ^G/G^) and littermate control (A/A, *Bcs1l* ^A/A^) mice. Columns (i.e. matched pairs) are reordered by hierarchical clustering (HCA, Ward aggregation method) using the age group (14, 24 and more than 30 days, >30) to label the tree leaves. Metabolites are grouped according to chemical classification. (B) Explicit fold changes with their corresponding 95% confidence intervals for all the carbohydrate intermediates measured in the three age groups. Significance levels: +, q<0.2; **, q<0.01 and ***, q<0.001. DHAP+3-PGA: mixture of dihydroxyacetonephosphate and 3-phosphoglyceraldehyde. (C) Periodic acid-Schiff staining of liver sections showing progressive glycogen depletion from 24 to 34 days old homozygotes.

**Figure 2:**
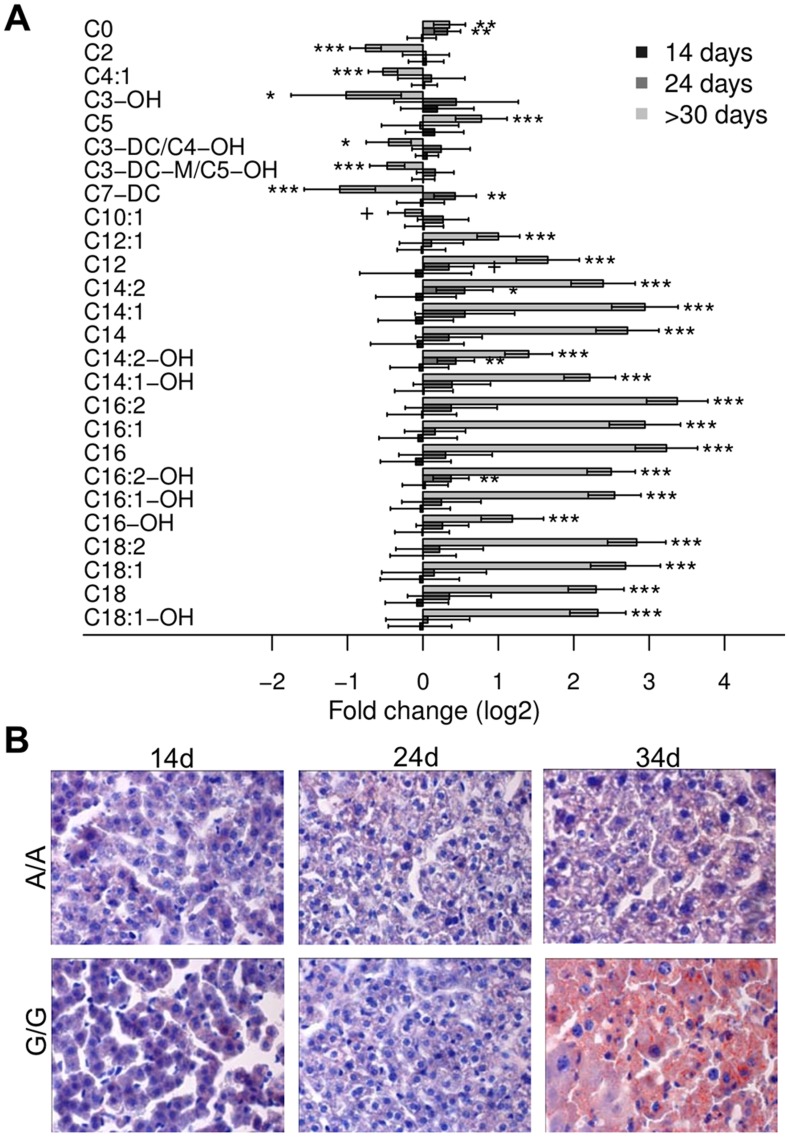
Acylcarnitines and lipid accumulation. (A) Log_2_ fold changes of the acylcarnitines found significant at a false discovery rate <0.1 in the three age groups (14, 24 and >30 days) between matched pairs of homozygous (G/G, *Bcs1l* ^G/G^) and control (A/A, *Bcs1l* ^A/A^) mice. Metabolites are presented in the increasing order of their molecular weight. Significance levels are defined as: +, q<0.2; *, q<0.05; **, q<0.01 and ***, q<0.001. (B) Oil-Red-O staining showing microvesicular lipid accumulation in hepatocytes of sick animal (34d), only.

**Figure 3:**
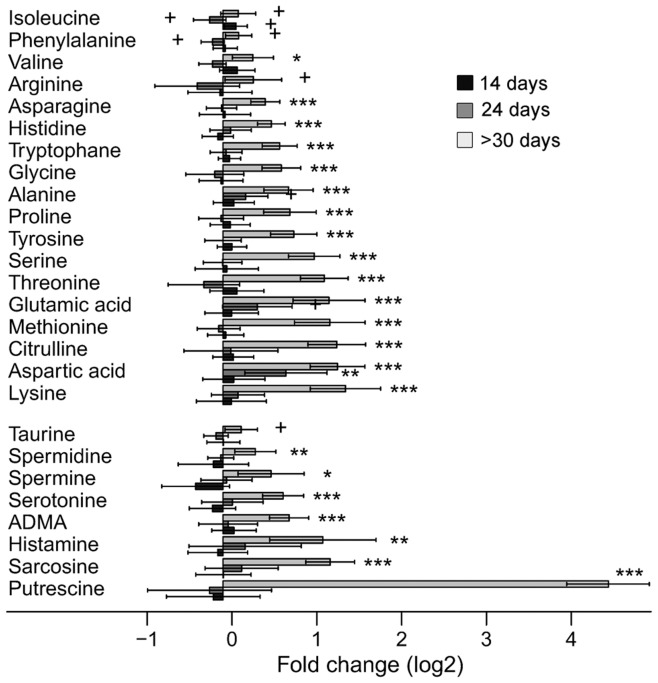
Amino acids and biogenic amines. (A) Log_2_ fold changes of amino acids and biogenic amines found significant at false discovery rate <0.1 in the three age groups (14, 24 and >30 days) between matched pairs of homozygous and control mice. Sick homozygotes (indicated as >30 days) have significantly elevated levels of the two metabolite groups, especially the cell signalling amine putrescine.

Comparing the pool of 198 detected metabolites to control animals, significant differences at FDR<0.1 were found both due to the mutation as such (102 compounds) and to the age of the animal (177 compounds). Using the same threshold, 141 metabolites exhibited positive interaction between the presence of the mutation and the age. A summary of the metabolites other than glycerophosphatidylcholines and sphingomyelins is given in a form of a heatmap in Fig. 1A (the latter groups are presented in Fig. S1). Explicit fold changes of the metabolites for which the impact of the mutation is age dependent (i.e. positive interaction at FDR<0.1) are presented separately (Fig. 1B, 2A, 3A). Amongst the 141 selected compounds, only one metabolite on day 14 (PC aa C42∶1, FC (log2) = −0.39, q<0.007) and 20 metabolites on day 24 (14 phosphatidylcholines, 5 acylcarnitines and aspartic acid, FC(log2) = −0.24 to 0.74, q<0.05) were significantly different (Fig. S1, Fig. 2A and 3A).

In an analysis of changes in sets of metabolites [18] glycerophosphatidylcholines (n = 91 metabolites) and compounds of energy metabolism (n = 9) were up-regulated on day 24 (*p*<0.05) whereas sphingomyelins (n = 14) and acylcarnitines (n = 41) were not significantly different (*p*<0.1 and *p*<0.2, respectively). No positive association could be found on day 14 or for any other metabolite class on day 24.

The most marked findings were increased metabolite levels in liver tissue of the animals at the end stage of the disease, aged over 30 days; up to 4-fold changes for most amino acids (q<0.05 for 15 out of 21, Fig. 3A) and all detected bile acids (q<0.001, Fig. 1A, Fig. S1). However, several short/medium length acylcarnitines (Fig. 2A), components of the energy metabolism pathway (Fig. 1B), and lipids (Fig. S1) were decreased. At 24 days of age, when initial histopathological changes, ie glycogen depletion, appear (Fig. 1C), levels of succinate, fumarate (Fig. 1B), acylcarnitines (Fig. 2A), and aminoacids (Fig. 3A) were up-regulated compared to control animals.

### Impaired Energy Metabolism in *Bcs1l^G/G^* Mice

The initial change in carbohydrate metabolism in homozygotes aged 24 days was an increase in succinate (Fig. 1B) concomitant with the glycogen depletion seen in histological sections (Fig. 1C). In these animals, also increases in medium- and long-chain acylcarnitines were found (Fig. 2A). In sick animals, a 2- to 3-fold increase was found in most medium- and long-chain acylcarnitines, whereas most short-chain acylcarnitines were decreased (Fig. 2A) concomitant with a clear steatosis in liver histology (Fig. 2B). Hexoses and lactic acid were decreased, whereas adenosine monophosphate (AMP) and several glycogenic amino acids were increased. Bile acids were highly increased in sick animals (Fig. 1A).

### Disturbance of Amino Acid Balance

Clearly increased amino acids were found in sick animals aged over 30 days, with less prominent findings present already at 24 days in homozygotes (Fig. 3). None of the biogenic amines were increased at 24 days, but clear increases were present in sick animals, especially the 5-fold increase of putrescine (Fig. 3).

### Oxidative Stress and Signs of Inflammation only in End Stage Disease

**Figure 4:**
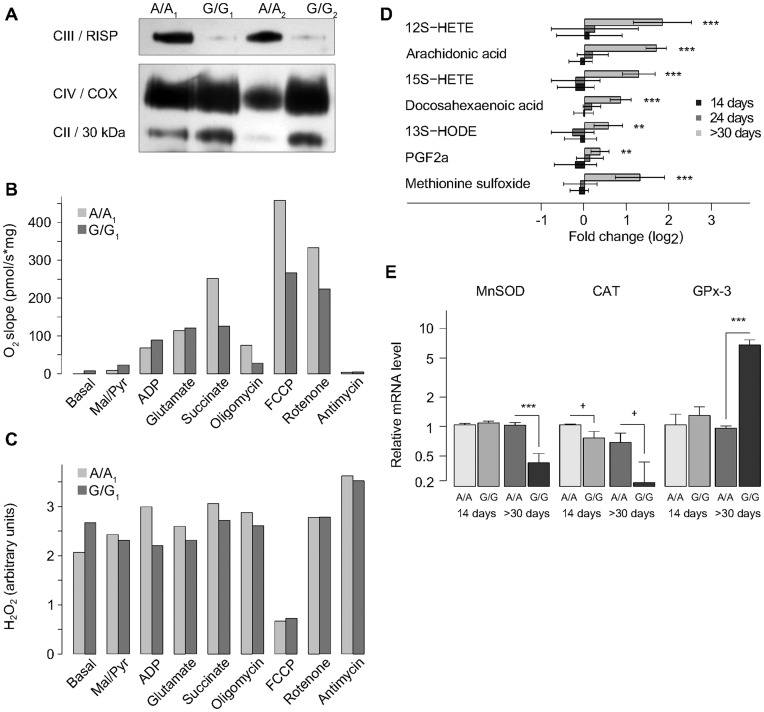
Respirometry and H_2_O_2_ production in mitochondria with RISP depleted CIII (A-C), and signs of oxidative stress in liver tissue (D, E). (A) Blue Native PAGE and Western blot showing decreased Rieske iron sulphur protein (RISP) incorporation in CIII in mitochondria from two representative *Bcs1l* ^G/G^ (G/G_1_, G/G_2_) mice compared to wild type (A/A_1_, A/A_2_) aged >30 d. (B) Respiratory chain oxygen consumption under convergent substrate input in CI and CII is impaired in G/G_1_. (C) Similar H_2_O_2_ production was detected with Amplex Red in isolated mitochondria of G/G_1_ and A/A_1_ mice. (D) In liver tissue homogenates of three age group animals, metabolomic analyses showed that log_2_ fold changes of the oxidative stress markers were significantly increased in sick animals only (>30 days). (E) Antioxidant defence in liver tissue measured with quantitative PCR in 14 days old and sick (>30 d) homozygotes expressed as relation to β-actin. In sick animals manganese superoxide dismutase (MnSOD) and catalase (Cat) were decreased, whereas glutathione peroxidase 3 (GPx-3) was increased.

In isolated mitochondria from sick *Bcs1l* ^G/G^ mice, lack of RISP in CIII (Fig. 4A) was accompanied with functional deficit in respiratory chain (Fig. 4B), but no differences in H_2_O_2_ production compared to littermate controls (n = 3 pairs Fig. 4C). Neither was any ROS effect found on mitochondrial proteins, ie the amount of carbonylated protein was similar in *Bcs1l* ^G/G^ mice aged over 30 days and littermate controls (n = 7 pairs, *Bcs1l* ^G/G^/*Bcs1l* ^A/A^ relative value, mean ± SD: 1.09±0.36).

In liver tissue, significant increases for methionine-sulfoxide and all detected prostaglandins (6 out of 15 that were screened for) were found in sick animals aged over 30 days (Fig. 4D). The antioxidant capacity of liver tissue, assessed on the basis of mRNA levels, revealed no differences in the expression of any of the antioxidant enzymes between homozygotes and controls aged 14 days (Fig. 4E). However, in end stage disease, expression of the mitochondrial antioxidant genes manganese superoxide dismutase and catalase was down-regulated, whereas the extracellular glutathione peroxidase 3 was up-regulated (Fig. 4E).

## Discussion

Since structural and functional CIII deficiencies develop progressively in the organs of *Bcs1l* ^G/G^ mice, with the liver being most affected [13] we investigated liver metabolites at three different ages; before any tissue changes, at disease onset, and when mice are clearly affected by the disease. The earliest metabolite change was a slightly decreased level of AMP, followed by increased levels of intermediates of the Krebs cycle concomitant with an alteration of acylcarnitine metabolism and increases in the amino acids alanine, glutamate, and aspartate, and in fatty acids. These metabolomic changes were accompanied by the first signs of tissue pathology as shown by glycogen depletion in livers from *Bcs1l* ^G/G^ mice.

In the end stage of the disease, the spectrum of metabolite changes was clearly different, demonstrating a liver deterioration with severe energy deficit, cellular degeneration, oxidative stress, and a starvation-like phenotype. The concomitant up-regulation of larger acylcarnitines, AMP, and a majority of the lipids suggest a decrease of the Krebs cycle activity, and a subsequent failure in anticipated switch in hepatocytes towards beta-oxidation in response to glycogen depletion. Decreased short-chain acylcarnitines indicate a limitation in beta-oxidation. A major deterioration in liver function is reflected in the increase of most amino acids and all detected bile acids.

**Figure 5:**
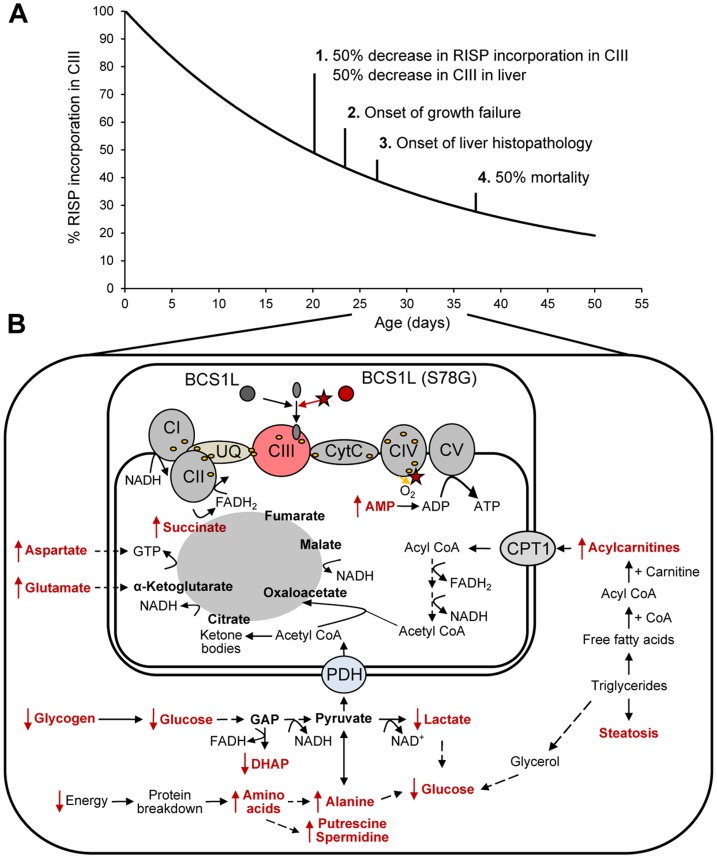
Hypothetic diagram of metabolic changes in hepatocytes in relation to disease progression in *Bcs1l* ^G/G^ mice. (A) With decreasing incorporation of Rieske iron sulphur protein (RISP) in complex CIII (CIII), the activity decreases and symptoms appear; at less than 50% incorporation growth failure starts. (B) Metabolomic changes in association with decreased CIII activity and respiratory chain dysfunction are depicted in red. Initial metabolic changes are elevated levels of succinate, aspartate and glutamate. Glycogen depletion leads to lack of glucose and lactate and induces beta-oxidation and protein breakdown resulting in accumulation of fatty acids and acylcarnitines as well as of the biogenic amines putrescine and spermidine. PDH. Pyruvate dehydrogenase complex, CPT1/2: Carnitine palmitoyl transferases 1 and 2. GAP, glyceraldehyde- 3 phosphate, DHAP, Dihydroxy acetone phosphate.

Under normal conditions, the liver ensures normoglycemia by glycogenolysis followed by increased gluconeogenesis [19]. During starvation, fatty acids are mobilized from adipose tissue, proteins are degraded and amino acids transported to the liver to serve as substrates for gluconeogenesis. Increased beta-oxidation and gluconeogenesis require energy forcing the respiratory chain to produce more adenosine triphosphate (ATP) [20]. This metabolic adaptation is compromised as CIII activity declines over time in *Bcs1l* ^G/G^ mice [13]. The observed glycogen depletion, transient increases in succinate and fumarate, persistent increases in medium- and long-chain acylcarnitines, and transient decrease, followed by accumulation of AMP are all consistent with this sequence of events (Fig. 5).

Low lactate content in the liver, despite increased blood levels [13], suggests that hepatocytes use it in gluconeogenesis. Maintaining glucose homeostasis is dependent on glycogenolysis and gluconeogenesis, which take place in liver and proximal tubules of the kidney. This mechanism seems to fail in sick *Bcs1l* ^G/G^ mice, in which liver and proximal tubules are the first and most affected organs with progressive CIII deficiency [13]. Thus, an explanation to liver being the most sensitive organ to CIII deficiency appears to be a failure of the respiratory chain in hepatocytes to respond to increased metabolic demands. In another mouse model with tissue-specific deletions of the transcription factor *Tfam*, respiratory chain deficiency seems to be successfully compensated by a switch from fatty acid oxidation to glycolysis, and by increased mitochondrial biogenesis, in myocardium [21] and in skeletal muscle [21], [22].

Energy starvation as well as respiratory chain deficiency may result in liver fat accumulation [23]. Liver fatty acid metabolism studies in mice under starvation demonstrated accumulation of triacylglycerol with similar changes in lysophosphatidylcholine and phosphatidylcholine profiles [24] as we identified in *Bcs1l* ^G/G^ mice. Phosphatidylcholines are essential for CIII activity since delipidation abolishes its function [25] indicating that enzyme-bound phospholipids play an important role for activity of respiratory chain complexes. The impact of respiratory chain failure on steatosis was investigated in preadipocytes incubated with the CIII inhibitor antimycin A. In these cells, accumulation of triglyceride vesicles concomitant with an elevated glucose uptake was present [26]. Likewise in sick *Bcs1l* ^G/G^ mice, microvesicular steatosis increases with decreasing CIII activity [13].

A recent study in tumour cells with a mitochondrial DNA mutation leading to lack of CIII activity showed that a glutamine-dependent reductive carboxylation, needing isocitrate dehydrogenase 1 and 2, can compensate for impaired mitochondrial oxidative metabolism [27]. Interestingly, acetyl CoA was mainly formed through this pathway and thus glutamine was the major precursor for synthesis of fatty acid. Increased intermediates were succinate and fumarate [27]. At disease onset we found increased levels of succinate and fumarate suggesting that such compensatory reductive carboxylation might occur, in addition to a possible signalling function of the intermediates [28] in *Bcs1l* ^G/G^ mice.

Liver samples at end stage disease in *Bcs1l* ^G/G^ mice contained elevated levels of the biogenic amino acids spermidine and putrescine, reflecting at least in part a limited capacity of the urea cycle. Both spermidine and putrescine play important roles in mediating cell death. Spermidine induces acetylation and deacetylation of nuclear and cytoplasmic proteins resulting in autophagy, removal of damaged cells and protein aggregates [29]. Biogenic amines are probably important for liver regeneration judging from a metabolomics study showing highly elevated tissue levels of putrescine and elevated levels of spermidine after partial hepatectomy [30].

Impaired respiratory chain function due to CIII deficiency may cause increased ROS production that together with liver steatosis contribute to tissue deterioration [14]. Increased ROS production has been found both in patient fibroblasts harbouring different BCS1L mutations [7], [17] and in mouse lung fibroblasts lacking RISP [16]. In contradiction, RISP inactivation by siRNA in hepatocyte and adipocyte cell cultures resulted in decreased levels of H_2_O_2_ [31], [32], [33].

In livers of *Bcs1l* ^G/G^ mice, ROS modified metabolites such as methionine sulfoxide and prostaglandins were detectable only at end stage disease. Further, antioxidant defence was similar in younger animals and changed only slightly in end stage disease compared to controls. In contrast to findings in fibroblasts harbouring other CIII deficiencies [7], [16], [17] no elevated H_2_O_2_ levels could be detected in liver mitochondria from *Bcs1l* ^G/G^ mice. Controversy prevails concerning mitochondrial ROS production. Generation of mitochondrial ROS varies depending on tissue and substrate characteristics [34]. There are *in vitro* studies of CIV and mtDNA deficiencies showing elevated ROS levels, whereas *in vivo* models do not confirm oxidative stress in tissue [35], [36], [37].

Oxidative stress is believed to be a contributing factor for disease phenotypes in disturbances of oxidative phosphorylation, including in development of steatosis. Cytosolic accumulation of fatty acids activates peroxisomal beta-oxidation and microsomal omega-oxidation resulting in ROS production [20]. Contradictory to our hypothesis, we did not find signs of increased oxidative stress at early time points. Only in the end stage disease, ROS modified metabolites such as methionine sulfoxide and prostaglandins were slightly increased in the metabolomics analysis, suggesting no major role of ROS in the onset of the disease.

Hepatocyte energy production is regulated by the energy sensors AMP kinase (AMPK) and members of the sirtuin family of NAD^+^ dependent deacetylases [38]. Decreased oxidative phosphorylation results in increased AMP/ATP and NAD^+^/NADH ratios thereby switching on catabolic pathways. AMPK activation in mitochondrial deficiencies is important for regulation of increased mitochondrial biogenesis. This was shown experimentally using an AMP precursor, AICAR, to correct complex IV deficiency in mouse muscle [39]. Furthermore, mice lacking the mitochondrial expressed Sirtuin 3 show signs of disturbed beta-oxidation under starvation such as accumulation of intermediates of fatty acid oxidation. In these mice, long-chain acyl CoA dehydrogenase was hyperacetylated resulting in reduced enzymatic activity and accumulation of long-chain acyl carnitines [40]. Long-chain acyl carnitines were accumulated in the sick *Bcs1l* ^G/G^ mice suggesting a possible disturbance in Sirtuin 3 activation.

In conclusion, using metabolomics we have shown that respiratory chain dysfunction in *Bcs1l* ^G/G^ mice due to CIII deficiency leads to a starvation-like condition with energy depletion. The findings suggest that the initial disturbances are decreased glycolysis and beta- oxidation concomitant with a decreased ATP production. Impaired mitochondrial metabolism cannot be corrected by eg. the proposed compensatory reductive carboxylation [27] and thus a *circulus vitiosus* occurs with exhausted energy supply and failing compensatory mitochondrial biogenesis to energy deprivation. Oxidative stress is not a central regulatory mechanism for disease onset, but may appear at the end stage of the disease. We envision that our mouse model can be used for further investigations on disease mechanisms in respiratory chain failure and for exploring interventions.

## Materials and Methods

### Mice

All animal experiments were performed with the approval of the Lund regional animal research ethic committee, Sweden (permits M170-06, M158-08, 31-8265/08). The *Bcs1l* c.232A>G mutation was introduced into mice using gene targeting [13]. Mice of mixed genetic background were maintained on rodent diet (Labfor R34, Lactamin, Stockholm, Sweden) and water was available ad libitum in a vivarium with 12 h light/dark cycle at 22°C. *Bcs1l* ^G/G^ mice of three age groups were studied; on postnatal day 14, when the animals are healthy (n = 14, nine males) and on day 24 (n = 8, all males), when initial histopathological findings appear, and on day 30 or more (median 34 days, range 30–43, n = 18, eleven males), when the animals are sick. The same numbers of littermate wild type mice (*Bcs1l* ^A/A^) or heterozygotes (*Bcs1l* ^A/G^) at each age were used as controls. Animals were sacrificed by cervical dislocation. Liver tissue samples were either immediately frozen on dry ice, fixed in 4% buffered formalin or used for the isolation of mitochondria [13]. The formalin-fixed tissues were embedded in paraffin according to normal pathology laboratory routine; slices were stained with hematoxylin-eosin for basic morphology and with periodic acid–Schiff with and without diastase for glycogen detection. From snap-frozen liver tissues standard Oil-Red-O fat staining was performed.

### Metabolomic Analysis

Frozen liver samples were weighed into 2 ml Precellys tubes (Peqlab Biotechnologie GmbH, Erlangen, Germany) equipped with ceramic beads. Tissues were homogenized using ethanol/dichloromethane (1/1) as extraction solvents [41]. Homogenates were centrifuged at 18000 *g* at 2°C for 5 min, the resulting supernatants were pipetted into a cryovial (1.5 ml, Biozym GmbH, Oldendorf, Germany) and analyzed immediately to avoid degradation of the analytes.

Flow injection analysis (FIA-MS/MS) based platform was employed for the simultaneous quantification of a broad range of endogenous intermediates, namely acylcarnitines, amino acids, sphingomyelins and glycerophospholipids. A chromatographic step (LC-MS/MS) was applied for the additional quantification of a series of amino acids and biogenic amines, small organic acids and other components from the energy metabolism pathway, bile acids, and eicosanoids. Samples were randomized on the plate prior to analysis to avoid potential confounding interaction between concentration and order of injection. Detailed experimental protocols have been published previously [41], [42]. FIA-MS/MS data were quantified with MetIQ software (integral part of Absolute*IDQ* kit p150, BIOCRATES Life Sciences AG, Innsbruck, Austria) and LC-MS/MS data were quantified with Analyst 1.4.2 software (Applied Biosystems, Darmstadt, Germany). All methods are validated for human plasma considering FDA Guidance for industry – Bioanalytical Method Validation. The initial pool of 253 measurements [42] was reduced to 198 by excluding 21 redundant analytes as well as 34 analytes for which detection rates were lower than 80%. In short, the final panel entering data analyses and interpretation consists of 41 acylcarnitines, 9 components of the energy metabolism, 21 amino acids, 11 biogenic amines, 6 bile acids, 6 prostaglandins, 91 glycerophosphatidylcholines, and 13 sphingomyelins. A comprehensive list of all metabolites that underwent further statistical treatment is provided in Table S1 alongside the adopted nomenclature for annotating metabolites.

### Statistical Analysis of Metabolomic Results

Measurement data were imported to the environment R (R Development Core Team, 2011, R: A language and environment for statistical computing. R Foundation for Statistical Computing, Vienna, Austria. ISBN 3-900051-07-0, URL http://www.R-project.org/.) for data sorting, statistical analyses and data presentation. All calculations were performed on the log basis 2 transformed concentration data with the non-detect imputed as previously published [43]. Adhering strictly to the initial experimental design, age and genotype effects and their interaction were estimated by general least squares regression. P values were obtained from likelihood ratio tests. Fold changes in log basis 2 were directly extracted from the linear model and presented with their 95% confidence intervals. To account for the large number of inferences being made, metabolites were initially selected on the basis of the false discovery rate (FDR) [44] and denoted as the FDR in the text. Adjustment [45] was adopted at individual metabolite level to control the family-wise error rate (denoted as q value, in the text). For testing whether set of metabolites, rather than individual compounds, were up-regulated at day 14 and 24 we applied a recently developed method [18] to each chemical class.

### Analysis of Respiratory Chain Assembly by Blue Native PAGE

Mouse liver mitochondria were isolated and processed for Blue Native PAGE and Western blot as described previously [13]. Respiratory chain complexes were identified with antibodies (MitoSciences, Eugene, Oregon, USA) detecting respiratory chain subunits CIII/RISP, CIV/COX and CII/30 kDa subunit, respectively.

### Analysis of Respiratory Chain Activity with High Resolution Respirometry

Respiratory chain function was analysed in isolated mitochondria incubated in MIR 05 (110 mM sucrose, 20 mM HEPES, 20 mM Taurine, 60 mM K- lactobionate, 3 mM MgCl_2_, 10 mM KH_2_PO_4_, pH 7,1) by high resolution respirometry using the substrate- uncoupler- inhibitor protocol [46] as described previously [13].

### Assessment of Hydrogen Peroxide by Amplex Red

Isolated mitochondria were incubated at 37°C in assay medium (MIR 05) supplemented with 4 µl horseradish peroxidase, superoxide dismutase and Amplex red according to the instructions of the manufacturer (Invitrogen, Carlsbad, CA, USA). Under these conditions, increased fluorescence indicates H_2_O_2_ release from mitochondria or superoxide released from mitochondria and converted to H_2_O_2_ by addition of superoxide dismutase. Fluorescence was measured after addition of substrate and inhibitors for the respiratory chain complexes in a Perkin Elmer fluorometer and analyzed with FL Winlab software. Increased fluorescence/time (relative units) is direct proportional to increased H_2_O_2_ release after subsequent substrate and inhibitor addition.

### Carbonylated Proteins

Protein oxidation by ROS was measured as carbonyl group production. Isolated mitochondria were incubated with DNP-hydrazine for 45 min at room temperature. The mixture was diluted with PBS, and 100 µl were used to coat a 96 well plate that was incubated over night at 4°C. The wells were washed three times with 0.9% NaCl+0.05% Tween-20. Then the plate was incubated with primary antibody, anti DNP (Invitrogen, Carlsbad, CA, USA), diluted 2000 times in incubation buffer (PBS, 0.1% BSA, 0.25% Tween 20) for 1 h incubation at 37°C, washed and incubated with secondary antibody, Swine-Anti-rabbit IgG-HRP, (Dako A/S; diluted 2000 times in incubation buffer) for 1 h at room temperature. At the end the plate was incubated with substrate solution (O-phenylenediamine, Dako S2045) according to the recommendations of the manufacturer. The extinction at 490 nm was measured on a spectrophotometer (1420 Multilabel counter VICTOR3**,** Perkin Elmer) [47], [48].

### RNA Preparation and Quantitative PCR to Assess Antioxidant Defense

Frozen liver tissue samples were placed in lysis buffer included in the RNeasy kit and homogenized in a TissueLyser (Qiagen, Hilden, Germany). RNA was extracted from the lysate using the RNeasy kit from Qiagen according to the instructions of the manufacturer and RNA quality was analyzed by agarose gel electrophoresis.

For cDNA synthesis 500 ng total RNA were reverse transcribed using the Taqman® reverse transcription reagents from Applied Biosystems and random hexamer primers included in the kit. The resulting cDNA was used as template in quantitative PCR on an Applied Biosystems StepOne cycler using Taqman Gene Expression assays from Applied Biosystems for the specific genes. The variation in replicates is shown as mean ±SD.

## Supporting Information

Figure S1 **Lipid metabolism.** Heatmap depicting the log basis 2 fold changes in lipid (rows) concentration determined in liver tissues between matched pairs (columns) of homozygote (G/G, *Bcs1l* ^G/G^) and control (A/A, *Bcs1l* ^A/A^) mice. Columns (i.e. matched pairs) are reordered by hierarchical clustering (HCA, Ward aggregation method) using the age group (14, 24 and 30+ days) to label the tree leaves. Metabolites are grouped according to the chemical classification employed in the manuscript. To facilitate the reading, fold changes are truncated at +/− 2 and light grey lines are drawn around the main groups highlighted by HCA and chemical classes.
(TIF)

Table S1 **Metabolite panel.** List of metabolites used in this study.
(DOC)

## References

[1] Ghezzi D, Arzuffi P, Zordan M, Da Re C, Lamperti C (2011). Mutations in TTC19 cause mitochondrial complex III deficiency and neurological impairment in humans and flies.. Nat Genet.

[2] Cruciat CM, Hell K, Folsch H, Neupert W, Stuart RA (1999). Bcs1p, an AAA-family member, is a chaperone for the assembly of the cytochrome bc(1) complex.. EMBO J.

[3] Nobrega FG, Nobrega MP, Tzagoloff A (1992). BCS1, a novel gene required for the expression of functional Rieske iron-sulfur protein in Saccharomyces cerevisiae.. EMBO J.

[4] Zara V, Palmisano I, Conte L, Trumpower BL (2004). Further insights into the assembly of the yeast cytochrome bc1 complex based on analysis of single and double deletion mutants lacking supernumerary subunits and cytochrome b.. Eur J Biochem.

[5] Benit P, Lebon S, Rustin P (2009). Respiratory-chain diseases related to complex III deficiency.. Biochim Biophys Acta.

[6] Diaz F, Kotarsky H, Fellman V, Moraes CT (2011). Mitochondrial disorders caused by mutations in respiratory chain assembly factors.. Semin Fetal Neonatal Med.

[7] Hinson JT, Fantin VR, Schonberger J, Breivik N, Siem G (2007). Missense mutations in the BCS1L gene as a cause of the Bjornstad syndrome.. N Engl J Med.

[8] Andreu AL, Hanna MG, Reichmann H, Bruno C, Penn AS (1999). Exercise intolerance due to mutations in the cytochrome b gene of mitochondrial DNA.. N Engl J Med.

[9] Morris AA, Taylor RW, Birch-Machin MA, Jackson MJ, Coulthard MG (1995). Neonatal Fanconi syndrome due to deficiency of complex III of the respiratory chain.. Pediatr Nephrol.

[10] de Lonlay P, Valnot I, Barrientos A, Gorbatyuk M, Tzagoloff A (2001). A mutant mitochondrial respiratory chain assembly protein causes complex III deficiency in patients with tubulopathy, encephalopathy and liver failure.. Nat Genet.

[11] Visapaa I, Fellman V, Vesa J, Dasvarma A, Hutton JL (2002). GRACILE syndrome, a lethal metabolic disorder with iron overload, is caused by a point mutation in BCS1L.. Am J Hum Genet.

[12] Fellman V, Rapola J, Pihko H, Varilo T, Raivio KO (1998). Iron-overload disease in infants involving fetal growth retardation, lactic acidosis, liver haemosiderosis, and aminoaciduria.. Lancet.

[13] Leveen P, Kotarsky H, Morgelin M, Karikoski R, Elmer E (2011). The GRACILE mutation introduced into Bcs1l causes postnatal complex III deficiency: a viable mouse model for mitochondrial hepatopathy.. Hepatology.

[14] Begriche K, Igoudjil A, Pessayre D, Fromenty B (2006). Mitochondrial dysfunction in NASH: causes, consequences and possible means to prevent it.. Mitochondrion.

[15] Rolo AP, Teodoro JS, Palmeira CM (2012). Role of oxidative stress in the pathogenesis of nonalcoholic steatohepatitis.. Free Radic Biol Med.

[16] Diaz F, Enriquez JA, Moraes CT (2012). Cells lacking Rieske iron-sulfur protein have a reactive oxygen species-associated decrease in respiratory complexes I and IV.. Mol Cell Biol.

[17] Moran M, Marin-Buera L, Gil-Borlado MC, Rivera H, Blazquez A (2010). Cellular pathophysiological consequences of BCS1L mutations in mitochondrial complex III enzyme deficiency.. Hum Mutat.

[18] Wu D, Lim E, Vaillant F, Asselin-Labat ML, Visvader JE (2010). ROAST: rotation gene set tests for complex microarray experiments.. Bioinformatics.

[19] Reddy JK, Rao MS (2006). Lipid metabolism and liver inflammation. II. Fatty liver disease and fatty acid oxidation.. Am J Physiol Gastrointest Liver Physiol.

[20] Browning JD, Horton JD (2004). Molecular mediators of hepatic steatosis and liver injury.. J Clin Invest.

[21] Hansson A, Hance N, Dufour E, Rantanen A, Hultenby K (2004). A switch in metabolism precedes increased mitochondrial biogenesis in respiratory chain-deficient mouse hearts.. Proc Natl Acad Sci U S A.

[22] Wredenberg A, Freyer C, Sandstrom ME, Katz A, Wibom R (2006). Respiratory chain dysfunction in skeletal muscle does not cause insulin resistance.. Biochem Biophys Res Commun.

[23] Fellman V, Kotarsky H (2011). Mitochondrial hepatopathies in the newborn period.. Semin Fetal Neonatal Med.

[24] van Ginneken V, Verhey E, Poelmann R, Ramakers R, van Dijk KW (2007). Metabolomics (liver and blood profiling) in a mouse model in response to fasting: a study of hepatic steatosis.. Biochim Biophys Acta.

[25] Wenz T, Hielscher R, Hellwig P, Schagger H, Richers S (2009). Role of phospholipids in respiratory cytochrome bc(1) complex catalysis and supercomplex formation.. Biochim Biophys Acta.

[26] Vankoningsloo S, Piens M, Lecocq C, Gilson A, De Pauw A (2005). Mitochondrial dysfunction induces triglyceride accumulation in 3T3-L1 cells: role of fatty acid beta-oxidation and glucose.. J Lipid Res.

[27] Mullen AR, Wheaton WW, Jin ES, Chen PH, Sullivan LB (2012). Reductive carboxylation supports growth in tumour cells with defective mitochondria.. Nature.

[28] He W, Miao FJ, Lin DC, Schwandner RT, Wang Z (2004). Citric acid cycle intermediates as ligands for orphan G-protein-coupled receptors.. Nature.

[29] Morselli E, Marino G, Bennetzen MV, Eisenberg T, Megalou E (2011). Spermidine and resveratrol induce autophagy by distinct pathways converging on the acetylproteome.. J Cell Biol.

[30] Jung YS, Kim SJ, Kwon DY, Kim YC (2011). Metabolomic analysis of sulfur-containing substances and polyamines in regenerating rat liver.. Amino Acids.

[31] Brunelle JK, Bell EL, Quesada NM, Vercauteren K, Tiranti V (2005). Oxygen sensing requires mitochondrial ROS but not oxidative phosphorylation.. Cell Metab.

[32] Patten DA, Lafleur VN, Robitaille GA, Chan DA, Giaccia AJ (2010). Hypoxia-inducible factor-1 activation in nonhypoxic conditions: the essential role of mitochondrial-derived reactive oxygen species.. Mol Biol Cell.

[33] Tormos KV, Anso E, Hamanaka RB, Eisenbart J, Joseph J (2011). Mitochondrial complex III ROS regulate adipocyte differentiation.. Cell Metab.

[34] Tahara EB, Navarete FD, Kowaltowski AJ (2009). Tissue-, substrate-, and site-specific characteristics of mitochondrial reactive oxygen species generation.. Free Radic Biol Med.

[35] Fukui H, Diaz F, Garcia S, Moraes CT (2007). Cytochrome c oxidase deficiency in neurons decreases both oxidative stress and amyloid formation in a mouse model of Alzheimer’s disease.. Proc Natl Acad Sci U S A.

[36] Kowaltowski AJ, de Souza-Pinto NC, Castilho RF, Vercesi AE (2009). Mitochondria and reactive oxygen species.. Free Radic Biol Med.

[37] Trifunovic A, Wredenberg A, Falkenberg M, Spelbrink JN, Rovio AT (2004). Premature ageing in mice expressing defective mitochondrial DNA polymerase.. Nature.

[38] Canto C, Auwerx J (2009). PGC-1alpha, SIRT1 and AMPK, an energy sensing network that controls energy expenditure.. Curr Opin Lipidol.

[39] Viscomi C, Bottani E, Civiletto G, Cerutti R, Moggio M (2011). In vivo correction of COX deficiency by activation of the AMPK/PGC-1alpha axis.. Cell Metab.

[40] Hirschey MD, Shimazu T, Goetzman E, Jing E, Schwer B (2010). SIRT3 regulates mitochondrial fatty-acid oxidation by reversible enzyme deacetylation.. Nature.

[41] Urban M, Enot DP, Dallmann G, Korner L, Forcher V (2010). Complexity and pitfalls of mass spectrometry-based targeted metabolomics in brain research.. Anal Biochem.

[42] Solberg R, Enot D, Deigner HP, Koal T, Scholl-Burgi S (2010). Metabolomic analyses of plasma reveals new insights into asphyxia and resuscitation in pigs.. PLoS One.

[43] Kim H, Golub GH, Park H (2005). Missing value estimation for DNA microarray gene expression data: local least squares imputation.. Bioinformatics.

[44] Benjamini Y, Hochberg Y (1995). Controlling the false discovery rate: a practical and powerful approach to multiple testing.. J R Stat Soc B.

[45] Holm S (1979). A simple sequentially rejective multiple test procedure.. Scand J of Statistics 6.

[46] Gnaiger E (2009). Capacity of oxidative phosphorylation in human skeletal muscle: new perspectives of mitochondrial physiology.. Int J Biochem Cell Biol.

[47] Buss H, Chan TP, Sluis KB, Domigan NM, Winterbourn CC (1997). Protein carbonyl measurement by a sensitive ELISA method.. Free Radic Biol Med.

[48] Olsson MG, Centlow M, Rutardottir S, Stenfors I, Larsson J (2010). Increased levels of cell-free hemoglobin, oxidation markers, and the antioxidative heme scavenger alpha(1)-microglobulin in preeclampsia.. Free Radic Biol Med.

