# Supplementary figures and images for: Metabolite Profiles Reveal Energy Failure and Impaired Beta-Oxidation in Liver of Mice with Complex III Deficiency Due to a BCS1L Mutation
Source: PLoS One. 2012 Jul 19;7(7):e41156. doi: 10.1371/journal.pone.0041156 (PMC3400604; doi:10.1371/journal.pone.0041156)

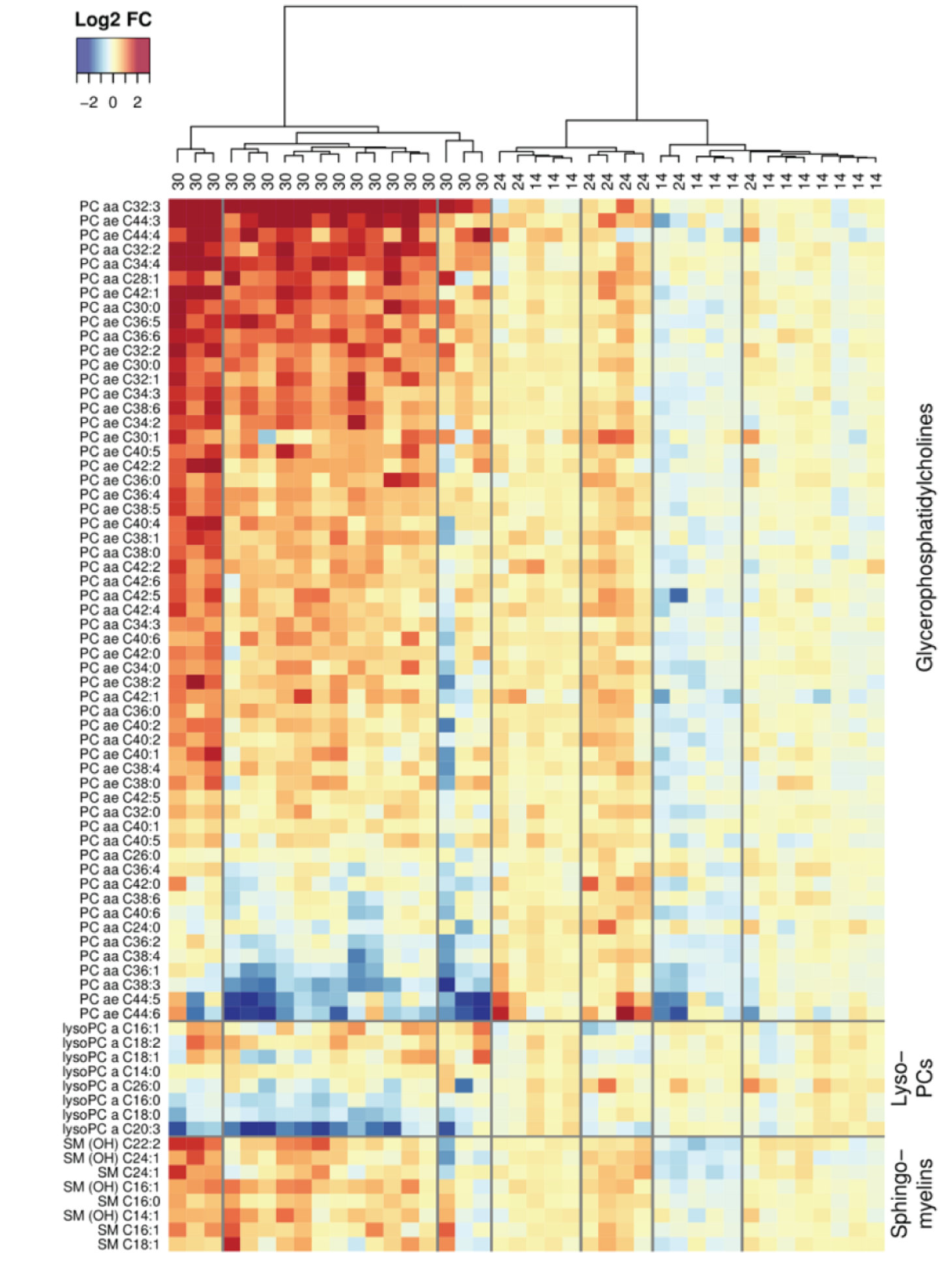

Supplement: Figure S1 — Lipid metabolism. Heatmap depicting the log basis 2 fold changes in lipid (rows) concentration determined in liver tissues between matched pairs (columns) of homozygote (G/G, Bcs1l G/G) and control (A/A, Bcs1l A/A) mice. Columns (i.e. matched pairs) are reordered by hierarchical clustering (HCA, Ward aggregation method) using the age group (14, 24 and 30+ days) to label the tree leaves. Metabolites are grouped according to the chemical classification employed in the manuscript. To facilitate the reading, fold changes are truncated at +/− 2 and light grey lines are drawn around the main groups highlighted by HCA and chemical classes. (TIF) [file pone.0041156.s001.tif]
