# Supplementary material for: Metabolite Profiles Reveal Energy Failure and Impaired Beta-Oxidation in Liver of Mice with Complex III Deficiency Due to a BCS1L Mutation
Source: PLoS One. 2012 Jul 19;7(7):e41156. doi: 10.1371/journal.pone.0041156 (PMC3400604; doi:10.1371/journal.pone.0041156)
Supplement: Table S1 — Metabolite panel. List of metabolites used in this study. (DOC) [file pone.0041156.s002.doc]

| **Acylcarnitines** | |
| --- | --- |
| C0: Carnitine (free) | C10:1: Decenoylcarnitine |
| C:2 Acetylcarnitine | C10:2: Decadienoylcarnitine |
| C3: Propionylcarnitine | C12: Dodecanoylcarnitine [Laurylcarnitine] |
| C3- DC/ C4OH: 3-Hydroxybutyrylcarnitine | C12-DC : Dodecanedioylcarnitine |
| C3-DC-M / C5-OH: 3-Hydroxyiso-valerylcarnitine / 3-Hydroxy-2-methylbutyryl | C12:1: Dodecenoylcarnitine |
| C3-DC-M / C5-OH: Hydroxypropionylcarnitine | C14: Tetradecanoylcarnitine [Myristylcarnitine] |
| C3:1: Propenoylcarnitine | C14:1: Tetradecenoylcarnitine [Myristoleylcarnitine] |
| C4: Butyrylcarnitine / Isobutyrylcarnitine | C14:1-OH : 3-Hydroxytetradecenoylcarnitine [3-Hydroxymyristoleylcarnitine] |
| C4:1: Butenoylcarnitine | C14:2: Tetradecadienoylcarnitine |
| C4:1-DC/C6: Hexanoylcarnitine [Caproylcarnitine] | C14:2-OH : 3-Hydroxytetradecadienoylcarnitine |
| C5: Isovalerylcarnitine / 2-Methylbutyryl-carnitine / Valerylcarnitine | C16: Hexadecanoylcarnitine [Palmitoylcarnitine] |
| C5-DC / C6-OH: Glutarylcarnitine | C16-OH : 3-Hydroxyhexadecanolycarnitine [3-Hydroxypalmitoylcarnitine] |
| C5-M-DC: Methylglutarylcarnitine | C16:1: Hexadecenoylcarnitine [Palmitoleylcarnitine] |
| C5:1:Tiglylcarnitine / 3-Methyl-crotonylcarnitine | C16:1-OH : 3-Hydroxyhexadecenoyl-carnitine [3-Hydroxypalmitoleylcarnitine] |
| C5:1-DC: Glutaconylcarnitine / Mesaconyl-carnitine (Undecanoylcarnitine ) | C16:2: Hexadecadienoylcarnitine |
| C6:1:Hexenoylcarnitine | C16:2-OH : 3-Hydroxyhexadecadienoylcarnitine |
| C7-DC: Pimelylcarnitine | C18: Octadecanoylcarnitine [Stearylcarnitine] |
| C:8: Octanoylcarnitine [Caprylylcarnitine] | C18:1: Octadecenoylcarnitine [Oleylcarnitine] |
| C8:1: Octenoylcarnitine | C18:1-OH : 3-Hydroxyoctadecenoylcarnitine [3-Hydroxyoleylcarnitine] |
| C9: Nonanoylcarnitine [Pelargonylcarnitine] | C18:2: Octadecadienoylcarnitine [Linoleylcarnitine] |
|  |  |
|  |  |
| **Biogenic amines** | |
| ADMA | Spermine |
| Hisatmine | Serotonine |
| Methionine- Sulfoxide | Phenylethylamine |
| Kynurenine | Taurine |
| Putrescine | Sarcosine |
| Spermidine |  |
|  |  |
|  |  |
| **Amino acids** | |
| Glycine | Glutamic acid |
| Alanine | Methionine |
| Serine | Histidine |
| Proline | Phenylalanine |
| Valine | Arginine |
| Threonine | Citrulline |
| Leucine | Tyrosine |
| Isoleucine | Tryptophane |
| Asparagine | Ornitihine |
| Aspartic acid | Lysine |
| Glutamine |  |
|  |  |
| **Energy metabolites** | |
| Lactic acid | Hexosephosphates |
| Fumaric acid | DHAP + 3- PGA (Dihydroxyacetonephosphate + 3-Phosphoglycerat) |
| Hexoses | cAMP (3’5’- cyclic Adenosine monophosphate) |
| Succinic acid | AMP (Adenosine-5’- monophosphate |
| Pentosephasphates |  |
|  |  |
|  |  |
| **Prostaglandines** | |
| 13S- HODE: 13(S)- hydroxyl- 9Z, 11 E- octadecadienoic acid | PGF2a: Prostaglandin F2 alpha |
| 12S- HETE: 12(S)- hydroxyl-5Z, 8Z, 10E, 14Z- eicosatetraenoic acid | Docosahexanoic acid |
| 15S- HETE: 15(S)- hydroxyl-5Z, 8Z, 11E, 13E- eicosatetraenoic acid | Arachidonic acid |
|  |  |
|  |  |
| **Bile acids** | |
| TCDCA: Taurochenodeoxycholic acid | TCA: Taurocholic acid |
| TLCA: Taurolithocholic acid | GCDCA: Glycochenodeoxycholic acid |
| GCA: Glycocholic acid | TUDCA: Tauroursdeoxycholic acid |
|  |  |
|  |  |
| **Sphingomyelines** | |
| SM (OH) C14:1 | SM (OH) C18:0 |
| SM (OH) C16:1 | SM (OH) C18:1 |
| SM (OH) C22:1 | SM (OH) C24:0 |
| SM (OH) C22:2 | SM (OH) C24:1 |
| SM (OH) C24:1 | SM (OH) C26:0 |
| SM (OH) C16:0 | SM (OH) C26:1 |
| SM (OH) C16:1 |  |
|  |  |
| **Lysophosphatidylcholines^1^** | |
| lysoPC a C14:0 | lysoPC a C20:4 |
| lysoPC a C16:0 | lysoPC a C24:0 |
| lysoPC a C16:1 | lysoPC a C26:0 |
| lysoPC a C17:0 | lysoPC a C26:1 |
| lysoPC a C18:0 | lysoPC a C28:0 |
| lysoPC a C18:1 | lysoPC a C28:1 |
| lysoPC a C18:2 | lysoPC a C6:0 |
| lysoPC a C20:3 |  |
|  |  |
|  |  |
| **Glycerophosphatidylcholines^2^** | |
| PC aa C42:0 | PC aa C34:1 |
| PC aa C42:1 | PC aa C34:2 |
| PC aa C42:2 | PC aa C34:3 |
| PC aa C42:4 | PC aa C34:4 |
| PC aa C42:5 | PC aa C36:0 |
| PC aa C42:6 | PC aa C36:1 |
| PC ae C30:0 | PC aa C36:2 |
| PC ae C30:1 | PC aa C36:3 |
| PC ae C30:2 | PC aa C36:4 |
| PC ae C32:1 | PC aa C36:5 |
| PC ae C32:2 | PC aa C36:6 |
| PC ae C34:0 | PC aa C38:0 |
| PC ae C34:1 | PC aa C38:3 |
| PC ae C34:2 | PC aa C38:4 |
| PC ae C34:3 | PC aa C38:5 |
| PC ae C36:0 | PC aa C38:6 |
| PC ae C36:1 | PC aa C40:1 |
| PC ae C36:2 | PC aa C40:2 |
| PC ae C36:3 | PC aa C40:3 |
| PC ae C36:4 | PC aa C40:4 |
| PC ae C36:5 | PC aa C40:5 |
| PC ae C38:0 | PC aa C40:6 |
| PC ae C38:1 | PC ae C40:3 |
| PC ae C38:2 | PC ae C40:4 |
| PC ae C38:3 | PC ae C40:5 |
| PC ae C38:4 | PC ae C40:6 |
| PC ae C38:5 | PC ae C42:0 |
| PC ae C38:6 | PC ae C42:1 |
| PC ae C40:1 | PC ae C42:2 |
| PC ae C40:2 | PC ae C42:3 |
| PC aa C24:0 | PC ae C42:4 |
| PC aa C26:0 | PC ae C42:5 |
| PC aa C28:1 | PC ae C42:6 |
| PC aa C30:0 | PC ae C44:3 |
| PC aa C32:0 | PC ae C44:4 |
| PC aa C32:1 | PC ae C44:5 |
| PC aa C32:2 | PC ae C44:6 |

^1^ Lysophosphatidylcholines are further differentiated with respect to the presence of ester (a) and ether (e) bonds in the glycerol moiety, where a single letter (a or e) indicates a bond with only one fatty acid residue. For e.g. lysoPC a C14:0 denotes a monoglycerophospholipid with 14 carbons in the fatty acid side chain. (<http://www.hmdb.ca/metabolites/HMDB10379>)

^2^ Glycero-phospholipids are further differentiated with respect to the presence of ester (a) and ether (e) bonds in the glycerol moiety, where two letters (aa, or ae) denote that the first and the second position of the glycerol scaffold are bound to a fatty acid residue. For e.g. PC_ae_32:1 denotes a plasmalogen phosphatidylcholine with 32 carbons in the two fatty acid side chains and a single double bond in one of them.
